# CHA_2_DS_2_-VASc score as predictor of ischemic stroke in patients undergoing coronary artery bypass grafting and percutaneous coronary intervention

**DOI:** 10.1038/s41598-017-11923-5

**Published:** 2017-09-12

**Authors:** Yaohua Tian, Chenlu Yang, Hui Liu

**Affiliations:** 1 Department of Epidemiology and Biostatistics, School of Public Health, Peking University, No. 38 Xueyuan Road, 100191 Beijing, China; 2 Department of Maternal and Child Health, School of Public Health, Peking University, No. 38 Xueyuan Road, 100191 Beijing, China; 3 Medical Informatics Center, Peking University, No. 38 Xueyuan Road, 100191 Beijing, China; 4 National Healthcare Data Center, Affiliated to National Center for Medical Service Administration, No. 38 Xueyuan Road, 100191 Beijing, China

## Abstract

Ischemic stroke following coronary revascularization procedures remains one of the most potentially devastating complications. CHA_2_DS_2_-VASc score has been widely used for stroke risk stratification in AF patients. The aim of this nationwide study was to examine the association between the CHA_2_DS_2_-VASc score and ischemic stroke following coronary revascularization procedures. We identified patients undergoing coronary revascularization procedures, coronary artery bypass grafting (CABG) and percutaneous coronary intervention (PCI), using the electronic Hospitalization Summary Reports. Logistic regression models were applied to evaluate the association of CHA_2_DS_2_-VASc score with the risk of post-procedural ischemic stroke. We identified 54,714 patients undergoing CABG and 263,063 patients undergoing PCI from 2013 to 2015. The CHA_2_DS_2_-VASc score had a positive graded association with the risk of post-procedural ischemic stroke in both CABG and PCI (*P* for trend <0.001). The adjusted risk of post-procedural ischemic stroke increased by an estimated 122.4% (odds ratio [OR], 2.22; 95% confidence interval [CI], 2.11–2.35) and 34.7% (OR, 1.35; 95% CI, 1.31–1.39) for each additional 1 point in the CHA_2_DS_2_-VASc score in CABG and PCI, respectively. In conclusion, these findings suggested that CHA_2_DS_2_-VASc score was an independent predictor of the development of post-procedural ischemic stroke in patients undergoing CABG and PCI.

## Introduction

Cardiovascular disease is the leading cause of death in the world, as well as in China^1, 2^. Coronary heart disease (CHD) accounts for the greatest proportion of cardiovascular disease. Coronary artery bypass grafting (CABG) and percutaneous coronary intervention (PCI) is the mainstay of revascularization procedures for patients with CHD. It was estimated that more than 1 million coronary revascularization procedures are performed annually in the United States^3^. The prognoses of patients undergoing revascularization procedures have aroused increasingly more attention^4–6^. Despite the continuous improvements in operating skills, peri-procedural care and service and management ability, post-procedural ischemic stroke remains one of the most potentially devastating complications. A large randomized, controlled trial involving 4,752 patients undergoing CABG surgery recruited from 79 hospitals in 19 countries reported that 30-day incidence rate of stroke after off-pump and on-pump CABG was 1.0% and 1.1%, respectively^7^. A recent meta-analysis of 6 contemporary randomized control trials including 5,673 patients with stable coronary artery disease who underwent PCI indicated that the incidence rate of post-procedural stroke was 2.0% at a weighted mean follow up of 55 months^8^. Prevailing evidence demonstrated that post-procedural ischemic stroke is an important cause of increased length of hospital stay, significant morbidity and mortality, and increased health care costs^9–11^. Stroke prevention in cardiac procedure has been an initiative championed by national societies as an overall effort to improve quality of clinical care^12^. Pre-procedural stratification of patients has significant clinical implications in individual decision-making, treatment selection, and post-procedural care.

CHA_2_DS_2_-VASc (congestive heart failure, hypertension, age ≥75, diabetes mellitus, prior stroke or transient ischemic attack (TIA), vascular disease, age 65–74, female) score has been widely used for stroke risk stratification in patients with atrial fibrillation^13^. A handful of studies have linked higher CHA_2_DS_2_-VASc score with increased risk of postoperative stroke in patients undergoing CABG surgery^14–18^, while data is limited in its application in PCI. Patients undergoing PCI have been demonstrated to be at elevated risk for ischemic stroke^19^. A high CHA_2_DS_2_-VASc score has been reported to be predictive of thrombotic outcomes in patients with AF undergoing PCI^20^. However, the specific association between CHA_2_DS_2_-VASc score and ischemic stroke following PCI has, to our knowledge, not been studied before. Better understanding of this association is important because PCI and CABG have different risk profiles and survival trajectories^6, 21^.

The aim of the present study, which was based on a multicenter national database, was to determine the association between CHA_2_DS_2_-VASc score and post-procedural ischemic stroke in patients undergoing CABG and PCI.

## Methods

### Data collection

Data used in this study were obtained from the electronic Hospitalization Summary Reports (HSRs) in the top-ranked public hospitals in care safety and quality as evaluated by the National Hospital Performance Evaluation Project in the National Healthcare Data Center. The hospital ranking system considers several aspects, including hospital infrastructure, medical service and management, technical level and efficiency, and quality and safety of clinical care. The information recorded on the HSR includes basic demographics, dates of admission and discharge, hospitalization and discharge diagnoses in Chinese and their corresponding International Classification of Diseases, 10th Revision (ICD-10) codes, treatment procedures and their corresponding International Classification of Diseases, 9th Revision, Clinical Modification (ICD-9-CM) codes, discharge status (survival status, drug allergy, and hospitalization infection), and financial costs. The present study is considered exempt from institutional review board approval since the data used was collected for administrative purpose without any personal identifiers.

An updated electronic HSR was implemented in 2012. The updated HSR contains up to 11 listed ICD-10 coding discharge diagnoses with the first one recorded designated as the principal diagnosis or primary illness, while the others are available for comorbid conditions and complications. The updated HSR also contains a maximum of 10 ICD-9-CM coding procedures. A new variable is assigned next to each listed diagnosis to specify the timing of diagnosis.

**Figure 1:**
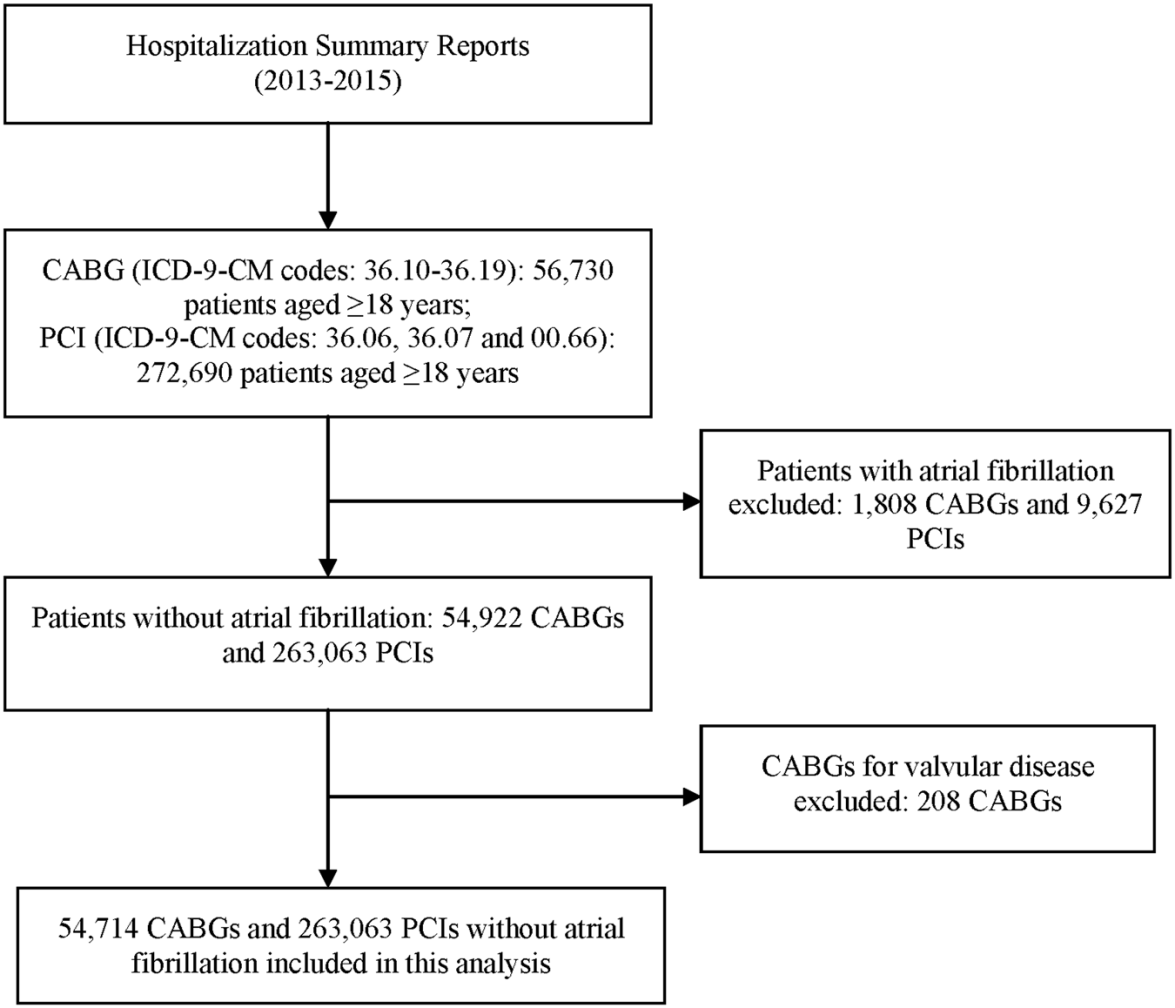
Flow chart of study enrollment.

In the present study, we identified patients aged ≥18 years undergoing CABG (ICD-9-CM procedure codes: 36.10–36.19) and PCI (ICD-9-CM procedure codes: 36.06, 36.07 and 00.66) between January 1, 2013 and December 31, 2015. Patients with urgent/emergent CABG or PCI were not included in our study. Patients with atrial fibrillation were excluded from this study. We also excluded procedures for valvular disease. To minimize the influence of coding inaccuracy, we used the corresponding Chinese terms to check the identified cases. The flow chart of the enrollment of the study population is shown in Fig. 1. In total, we identified 54,714 patients undergoing CABG and 263,063 patients undergoing PCI in 172 hospitals in 24 provinces across China.

### Measurements

The primary outcome in this study, post-procedural ischemic stroke, was defined as any ischemic stroke occurring in the period between the beginning of the procedure and the patient’s death or discharge from the hospital. Ischemic stroke was diagnosed according to the WHO criteria^22^ combined with brain computed tomography or magnetic resonance imaging confirmation.

The key independent variable in the present study was the CHA_2_DS_2_-VASc score. We calculated CHA_2_DS_2_-VASc score for each patient and 1 point was assigned for heart failure, hypertension, diabetes mellitus, vascular disease, age between 65 and 74 years and female gender whereas 2 points were assigned for ischemic stroke/TIA and age ≥75 years. Patients were classified into six groups according the following CHA_2_DS_2_-VASc scores: 0, 1, 2, 3, 4, and ≥5.

### Statistical analysis

In this study, categorical variables are reported as proportion (%) whereas numerical data are reported as mean ± standard deviation (SD). The incidence rates of post-procedural ischemic stroke between different CHA_2_DS_2_-VASc score groups were compared using Pearson’s χ^2^ test. We applied the Cochran–Armitage test for trend to analyze the association between the occurrence of post-procedural ischemic stroke and CHA_2_DS_2_-VASc score. To allow the adjustment for other comorbid conditions, association rule mining model was applied to identify comorbidities associated with post-procedural ischemic stroke^23^. Finally, a history of hyperlipidemia, chronic obstructive pulmonary disease (COPD), ischemic heart disease, AF and previous cardiac and vascular implants and grafts were significantly associated with post-procedural ischemic stroke (Figure S1 and S2). Univariable and multivariable logistic regression models were used to measure the odds ratios (ORs) and 95% confidence intervals (95% CIs) for post-procedural ischemic stroke associated with CHA_2_DS_2_-VASc score. The multivariable model was simultaneously adjusted for hyperlipidemia, COPD, ischemic heart disease, AF and previous cardiac and vascular implants and grafts. The C-statistic, which measures the area under the receiver operating characteristic (ROC), was used to evaluate the predictive ability of CHA_2_DS_2_-VASc score in the risk of postoperative stroke. All reported *P* values were nominal and two-sided. All statistical analyses were performed by R software.

## Results

Table 1 shows the demographic characteristics of the 54,714 patients undergoing CABG and 263,063 patients undergoing PCI by the presence of post-procedural ischemic stroke. Patients with post-procedural ischemic stroke had a higher prevalence of comorbid conditions and a higher CHA_2_DS_2_-VASc score.

**Table 1:** Demographic characteristics of patients undergoing coronary artery bypass grafting (CABG) and percutaneous coronary intervention (PCI).

Variable	CABG (N = 54714)			PCI (N = 263063)		
	Stroke (N = 612)	No stroke (N = 54102)	*P*	Stroke (N = 1874)	No stroke (N = 261189)	*P*
Age, (year) (mean ± SD)	63.4 ± 7.8	61.7 ± 9.6	0.006	65.6 ± 9.7	61.8 ± 10.9	<0.001
≤64 (%)	333 (54.4)	32700 (60.4)		871 (46.5)	155210 (59.4)	
65–74 (%)	232 (37.9)	17334 (32.0)		660 (35.2)	71922 (27.5)	
≥75 (%)	47 (7.7)	4068 (7.5)		343 (18.3)	34057 (13.0)	
Sex			0.177			<0.001
Men (%)	448 (73.2)	40881 (75.6)		1234(65.8)	190642 (73.0)	
Women (%)	164 (26.8)	13221 (24.4)		640 (34.2)	70547 (27.0)	
Hypertension (%)	457 (74.7)	30224 (55.9)	<0.001	1351(72.1)	153692 (58.8)	<0.001
Diabetes mellitus (%)	257 (42.0)	16535 (30.6)	<0.001	631 (33.7)	75858 (29.0)	<0.001
Heart failure (%)	183 (29.9)	19177 (35.4)	0.004	740 (39.5)	79800 (30.6)	<0.001
Vascular disease (%)	110 (18.0)	4027 (7.4)	<0.001	456 (24.3)	27218 (10.4)	<0.001
Ischemic stroke/TIA (%)	405 (66.2)	1122 (2.1)	<0.001	108 (5.8)	8770 (3.4)	<0.001
Hyperlipidemia (%)	280 (45.8)	11726 (21.7)	<0.001	431 (23.0)	54836 (21.0)	0.034
COPD (%)	0	263 (0.5)	0.084	25 (1.3)	3419 (1.3)	0.924
Ischemic heart disease (%)	509 (83.2)	38315 (70.8)	<0.001	1432(76.4)	183418 (70.2)	<0.001
Previous cardiac and vascular implants and grafts	66 (10.8)	4305 (8.0)	0.010	252 (13.4)	50466 (19.3)	<0.001
CHA_2_DS_2_-VASc score						
Mean ± SD	3.77 ± 1.56	2.05 ± 1.32	<0.001	2.87 ± 1.43	2.16 ± 1.46	<0.001
0 (%)	14 (2.3)	5899 (10.9)	<0.001	73 (3.9)	30488 (11.7)	<0.001
1 (%)	37 (6.0)	14186 (26.2)		222 (11.8)	65473 (25.1)	
2 (%)	65 (10.6)	15693 (29.0)		495 (26.4)	68095 (26.1)	
3 (%)	141 (23.0)	10760 (19.9)		510 (27.2)	49978 (19.1)	
4 (%)	156 (25.5)	5357 (9.9)		323 (17.2)	29205 (11.2)	
≥5 (%)	199 (32.5)	2207 (4.1)		251 (13.4)	17948 (6.9)	

SD = standard deviation; TIA = transit ischemic stroke; COPD = chronic obstructive pulmonary disease.

Table 2 presents the post-procedural ischemic stroke occurrences according to CHA_2_DS_2_-VASc score. There were 612 (1.1%) and 1,874 (0.7%) ischemic stroke cases following CABG and PCI, respectively. Among patients undergoing CABG, the prevalence rate of post-procedural ischemic stroke increased steadily across CHA_2_DS_2_-VASc score groups, ranging from 0.2% among patients with CHA_2_DS_2_-VASc score of 0 to 0.3% among patients with CHA_2_DS_2_-VASc score of 1, 0.4% among patients with CHA_2_DS_2_-VASc score of 2, 1.3% among patients with CHA_2_DS_2_-VASc score of 3, 2.8% among patients with CHA_2_DS_2_-VASc score of 4, and 8.3% among patients with CHA_2_DS_2_-VASc score ≥ 5. The prevalence rates of post-procedural ischemic stroke in patients undergoing PCI with CHA_2_DS_2_-VASc score of 0, 1, 2, 3, 4 and ≥5 were 0.2%, 0.3%, 0.7%, 1.0%, 1.1% and 1.4%, respectively. The values of rho Spearman of correlation of CHA_2_DS_2_-VASc and ischemic stroke in CABG and PCI were 0.111 (*P* < 0.001) and 0.074 (*P* < 0.001), respectively.

**Table 2:** Post-procedural ischemic stroke occurrence in patients undergoing coronary artery bypass grafting (CABG) and percutaneous coronary intervention (PCI) stratified by CHA_2_DS_2_-VASc score.

	Total	Event (%)	Spearman’s rho	*P*-value
CABG	54,714	612 (1.1)	0.111	<0.001
CHA_2_DS_2_-VASc score				
0	5,913	14 (0.2)		
1	14,223	37 (0.3)		
2	15,758	65 (0.4)		
3	10,901	141 (1.3)		
4	5,513	156 (2.8)		
≥5	2,406	199 (8.3)		
PCI	272,690	1,874 (0.7)	0.074	<0.001
CHA_2_DS_2_-VASc score				
0	30,561	73 (0.2)		
1	65,695	222 (0.3)		
2	68,590	495 (0.7)		
3	50,488	510 (1.0)		
4	29,528	323 (1.1)		
≥5	18,199	251 (1.4)		

Table 3 shows the association between CHA_2_DS_2_-VASc score and the risk of post-procedural ischemic stroke. There was a positive graded association between CHA_2_DS_2_-VASc score and post-procedural ischemic stroke following both CABG and PCI (*P* for trend <0.001). The adjusted risk of post-procedural ischemic stroke increased by an estimated 122.4% (odds ratio [OR], 2.22; 95% confidence interval [CI], 2.11–2.35) and 34.7% (OR, 1.35; 95% CI, 1.31–1.39) for each additional 1 point in the CHA_2_DS_2_-VASc score in CABG and PCI, respectively (data was not shown in tables). As compared to patients undergoing CABG with a CHA_2_DS_2_-VASc score of 0, the corresponding adjusted ORs of post-procedural ischemic stroke were 1.03 (95% CI: 0.56–1.91) and 6.28 (95% CI: 3.69–10.68) for those with a CHA_2_DS_2_-VASc score of 1, and ≥2, respectively. The corresponding values in patients undergoing PCI were 1.42 (95% CI: 1.09–1.85) and 4.04 (95% CI: 3.19–5.11), respectively (Table S1). The C-statistic values estimated by CHA_2_DS_2_-VASc score in CABG and PCI were 0.796 (95% CI: 0.778–0.815, *P* < 0.001) and 0.640 (95% CI: 0.628–0.651, *P* < 0.001), respectively (Figure S3).

**Table 3:** Adjusted odds ratios (ORs) of post-procedural ischemic stroke in patients undergoing coronary artery bypass grafting (CABG) and percutaneous coronary intervention (PCI) stratified by CHA_2_DS_2_-VASc score.

	Crude OR	95% CI	Adjusted OR^b^	95% CI
CABG				
0	1			
1	1.10	0.59–2.04	1.03	0.56–1.91
2	1.75	0.98–3.11	1.57	0.88–2.81
3	5.52^a^	3.19–9.58	4.98^a^	2.87–8.64
4	12.27^a^	7.09–21.23	11.17^a^	6.45–19.34
≥5	37.99^a^	22.05–65.47	35.34^a^	20.49–60.97
*P* value for trend	<0.001			
PCI				
0	1			
1	1.42^a^	1.09–1.85	1.42^a^	1.09–1.85
2	3.04^a^	2.37–3.88	3.06^a^	2.40–3.92
3	4.26^a^	3.33–5.45	4.33^a^	3.38–5.53
4	4.62^a^	3.58–5.96	4.70^a^	3.64–6.07
≥5	5.84^a^	4.50–7.59	5.92^a^	4.56–7.69
*P* value for trend	<0.001			

^a^ *P* < 0.001. ^b^Adjusted for a history of hyperlipidemia, chronic obstructive pulmonary disease, ischemic heart disease, and previous cardiac and vascular implants and grafts. OR = odds ratio; 95% CI = 95% confidence interval.

Table 4 shows the association between post-procedural ischemic stroke and the risk of in-hospital mortality. In CABG, the average days between the CABG and the mortality events for patients with and without post-procedural stroke were 48.1 and 17.3 days, respectively. In PCI, the corresponding values were 10.7 and 6.8 days, respectively. Post-procedural ischemic stroke was significantly associated with in-hospital mortality in PCI, but not in CABG after adjustment for CHA_2_DS_2_-VASc score and other potential confounders.

**Table 4:** Post-procedural ischemic stroke and in-hospital mortality in patients undergoing coronary artery bypass grafting (CABG) and percutaneous coronary intervention (PCI).

	Death (%)	Crude OR	95% CI	Adjusted OR^a^	95% CI
CABG	921 (1.7)				
No-stroke patients	908 (1.7)	1		1	
Stroke patients	13 (2.1)	1.27	0.73–2.21	1.06	0.60–1.86
PCI	1,355 (0.5)				
Stroke patients	23 (1.2)	1		1	
No-stroke patients	1,332 (0.5)	2.42^b^	1.60–3.67	1.81^c^	1.20–2.75

^a^Adjusted for CHA_2_DS_2_-VASc score, a history of hyperlipidemia, chronic obstructive pulmonary disease, ischemic heart disease, and previous cardiac and vascular implants and grafts. ^b^ *P* < 0.001. ^c^ *P* < 0.05. OR = odds ratio; 95% CI = 95% confidence interval.

## Discussion

In this study from a national database identifying 54,714 CABGs and 263,063 PCIs between 2013 and 2015 in China, CHA_2_DS_2_-VASc score was positively associated with the risk of post-procedural ischemic stroke. This is the first study, to our knowledge, to simultaneously evaluate the CHA_2_DS_2_-VASc score in both CABG and PCI. Epidemiologic, quality evaluation, and health services studies aimed at improving the health outcomes of patients undergoing CABG and PCI are gaining increasingly more attention^4–6^, CHA_2_DS_2_-VASc score promises to be a highly useful tool in such research.

The prevention of post-procedural ischemic stroke has significant clinical implications because of its significant impacts on prognoses of patients after primary treatment^9–11^. Several risk stratification schemes have been proposed to stratify risk of post-procedural stroke, such as the Multicenter Study of Perioperative Ischemia Research Group (McSPI) and the Northern New England Cardiovascular Disease Study Group (NNECDSG) scores^24, 25^. However, none has been widely applied in routine clinical practice. CHA_2_DS_2_-VASc score, which was initially employed as a risk assessment tool for predicting stroke in patients with AF, has been extensively used in clinical practice and in some guidelines for treatment selection for stroke prevention^13, 26^. Recently, CHA_2_DS_2_-VASc score has been used to discriminate patients at high risk of stroke in patients undergoing CABG surgery^14–17^. In this study, we also observed a positive association between CHA_2_DS_2_-VASc score and post-procedural ischemic stroke following CABG. The simplicity and strong operability of CHA_2_DS_2_-VASc algorithm would be beneficial to ensure routine evaluation in the clinical setting. These findings suggest that the CHA_2_DS_2_-VASc score could be used as a complementary approach of stratifying the post-procedural ischemic stroke risk in patients undergoing CABG. The ability of CHA_2_DS_2_-VASc scores in predicting post-procedural ischemic stroke is of concern and provides a critical analysis for possible prevention strategies.

In this study, CHA_2_DS_2_-VASc score was significantly associated with the risk of post-procedural ischemic stroke following PCI. Although the predictive value of CHA_2_DS_2_-VASc score in predicting ischemic stroke following PCI is less established, the individual components of the CHA_2_DS_2_-VASc score have been demonstrated to represent significant risk factors for post-procedural ischemic stroke^27, 28^. For example, an analysis of 426,046 patients undergoing PCI in England and Wales between 2007 and 2012 in the British Cardiovascular Intervention Society (BCIS) database demonstrated that age, female gender, a history of stroke were significant predictors of post-procedural ischemic stroke/TIA, and ischemic stroke was independently associated with both 30-day mortality and in-hospital major adverse cardiovascular events (a composite of in-hospital mortality, myocardial infarction or reinfarction, and revascularization)^27^. An observational, multicenter, prospective study including 929 patients with AF indicated that a high CHA_2_DS_2_-VASc score was predictive of major adverse events (a composite of all-cause mortality, myocardial infarction, repeat revascularization, stent thrombosis, transient ischemic attack, stroke or other arterial thromboembolism) following PCI^20^. In our study, the use of ischemic stroke as the end point instead of a combined end point can better understand the association of CHA_2_DS_2_-VASc score with health outcomes following PCI.

The CHA_2_DS_2_-VASc score with risk estimates may possess some value in clinical practice. The CHA_2_DS_2_-VASc score could be used as a single index that reflect the overall burden of age, gender and comorbid conditions; this would diminish the confounding bias that results from these factors without necessitating the extremely large sample sizes that would be required to control for each condition separately^29^. A substantial confounding bias can be controlled if a strong risk factor could be accurately measured in health outcome studies^30^. Our study also illustrates the necessity to account for these factors. Patients undergoing CABG and PCI with a CHA_2_DS_2_-VASc score ≥5 had 40.5 and 6.0 times the risk of developing ischemic stroke, respectively, than those with a CHA_2_DS_2_-VASc score of 0. The CHA_2_DS_2_-VASc score can also be used to classify patients into risk categories or levels, leading to more tailored approaches for patient management. It may be useful in helping physicians to determine what treatment option would best suit a specific patient. In addition, using the CHA_2_DS_2_-VASc score as a screening tool, patients could be cared for in a suitable setting. Patients with a high CHA_2_DS_2_-VASc score suggesting a significant risk of post-procedural ischemic stroke would receive a high standard of medical care. Therefore, the CHA_2_DS_2_-VASc should be taken into consideration when planning individual treatment course. Research indicate that comorbid conditions had substantial influences on health outcomes and quality of life among cardiovascular patients after primary treatment^31^. Therefore, the CHA_2_DS_2_-VASc algorithm can be used as a supplementary factor to consider when managing follow-up care after primary treatment. Studies that examine these and other possible clinical applications of the CHA_2_DS_2_-VASc algorithm should be conducted to improve clinical practice.

Unique features of this study include the large sample size, national multicenter design, and real world data on both CABG and PCI procedures, all of which increase the generalizability of findings. However, our study has a few noteworthy limitations. First, the retrospective data collection and analysis may have introduced some confounding bias. Another limitation was our inability to account for the influences caused by patient- and procedure-related characteristics, such as smoking status, dietary habits and physical activity. In addition, each diagnosis of disease and procedure in this study was made by ICD codes, which may cause bias from coding errors. However, the validity and reliability of this database have been proved in prior studies^32, 33^. To minimize such potential confounding bias, we used the corresponding Chinese terms to check the identified diseases and procedures. Antithrombotic therapy may affect the development of post-procedural ischemic stroke. However, data on antithrombotic therapies was not available in our database. An additional analysis stratified on the use of oral anticoagulant drugs should be performed in the future study. Finally, it could be useful to compare with GRACE score or other surgical risk scores to predict stroke. However, as data on several components of these scores, such as creatinine level and left ventricular ejection fraction, was not available in our database, we cannot calculate these scores for each patient. Future studies are needed to evaluate the performance of these scores in the prediction of post-procedural ischemic stroke.

The present study is the first to simultaneously evaluate the CHA_2_DS_2_-VASc score in both CABG and PCI. The risk of post-procedural ischemic stroke increased with a high CHA_2_DS_2_-VASc score. Future studies should be conducted to test the performance and clinical application of the CHA_2_DS_2_-VASc score in various databases.

## Electronic supplementary material

Supplementary information
