# Supplementary material for: CHA2DS2-VASc score as predictor of ischemic stroke in patients undergoing coronary artery bypass grafting and percutaneous coronary intervention
Source: Sci Rep. 2017 Sep 12;7:11404. doi: 10.1038/s41598-017-11923-5 (PMC5595984; doi:10.1038/s41598-017-11923-5)
Supplement: Supplementary file 1 — Supplementary information [file 41598_2017_11923_MOESM1_ESM.pdf]

Supplementary materials

CHA_2DS_2-VASc score as predictor of ischemic stroke in patients undergoing coronary artery bypass grafting and percutaneous coronary intervention

Yaohua Tian[1] · Chenlu Yang[2] · Hui Liu[3,4,*]

[1] Department of Epidemiology and Biostatistics, School of Public Health, Peking University, No.38 Xueyuan Road, 100191 Beijing, China

[2] Department of Maternal and Child Health, School of Public Health, Peking University, No.38 Xueyuan Road, 100191 Beijing, China

[3] Medical Informatics Center, Peking University, No.38 Xueyuan Road, 100191 Beijing, China

[4] National Healthcare Data Center, Affiliated to National Center for Medical Service Administration, No.38 Xueyuan Road, 100191 Beijing, China

[*] **Corresponding author**: Hui Liu, Medical Informatics Center, Peking University, No.38 Xueyuan Road, 100191 Beijing, China or National Healthcare Data Center, Affiliated to National Center for Medical Service Administration, No.38 Xueyuan Road, 100191 Beijing, China.

Phone: +86-010-82805907 Fax: +86-010-82805901

E-mail address: ymauil@bjmu.edu.cn (H. Liu).

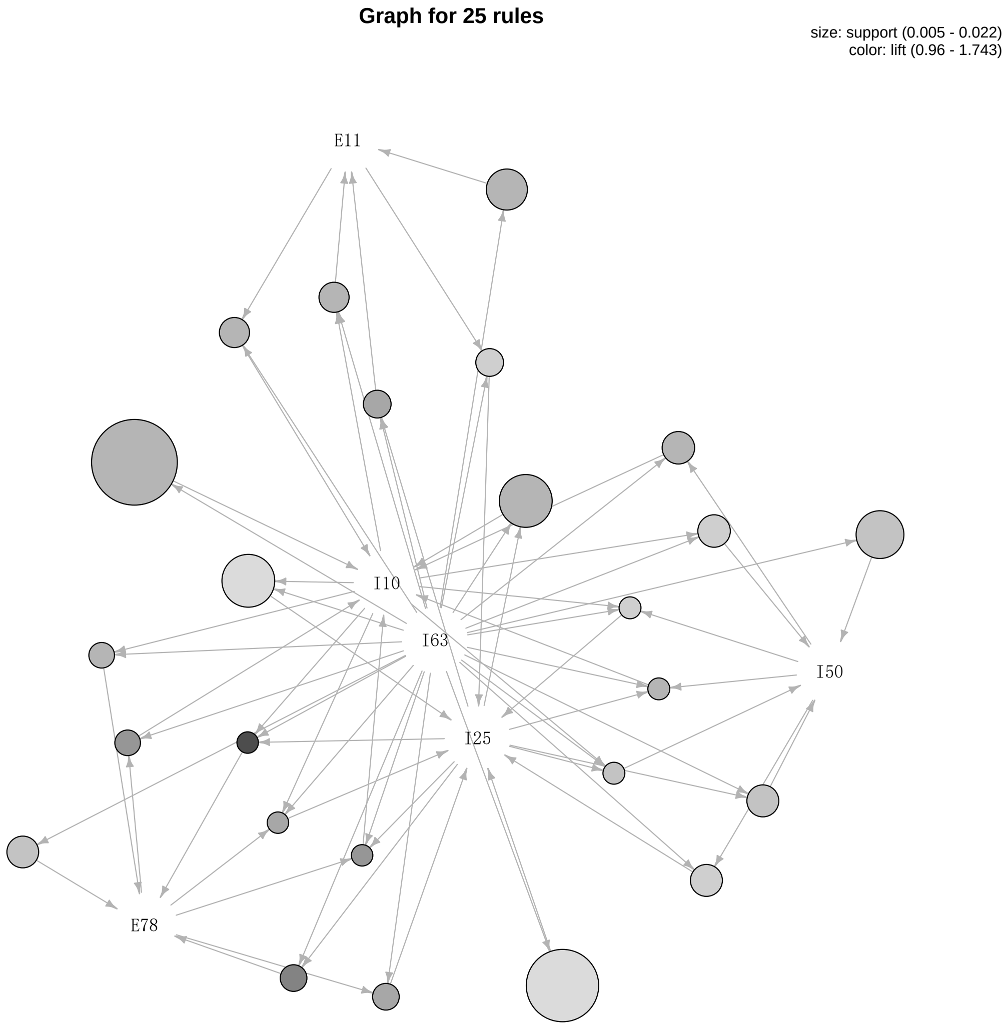

Supplementary figure S1 Identification of comorbid conditions associated with post-procedural ischemic stroke following coronary artery bypass grafting based on association rule mining.

I63: post-procedural ischemic stroke; I10: hypertension; I25: ischemic heart disease; I50: congestive heart failure; E78: hyperlipidemia; E11: diabetes mellitus.

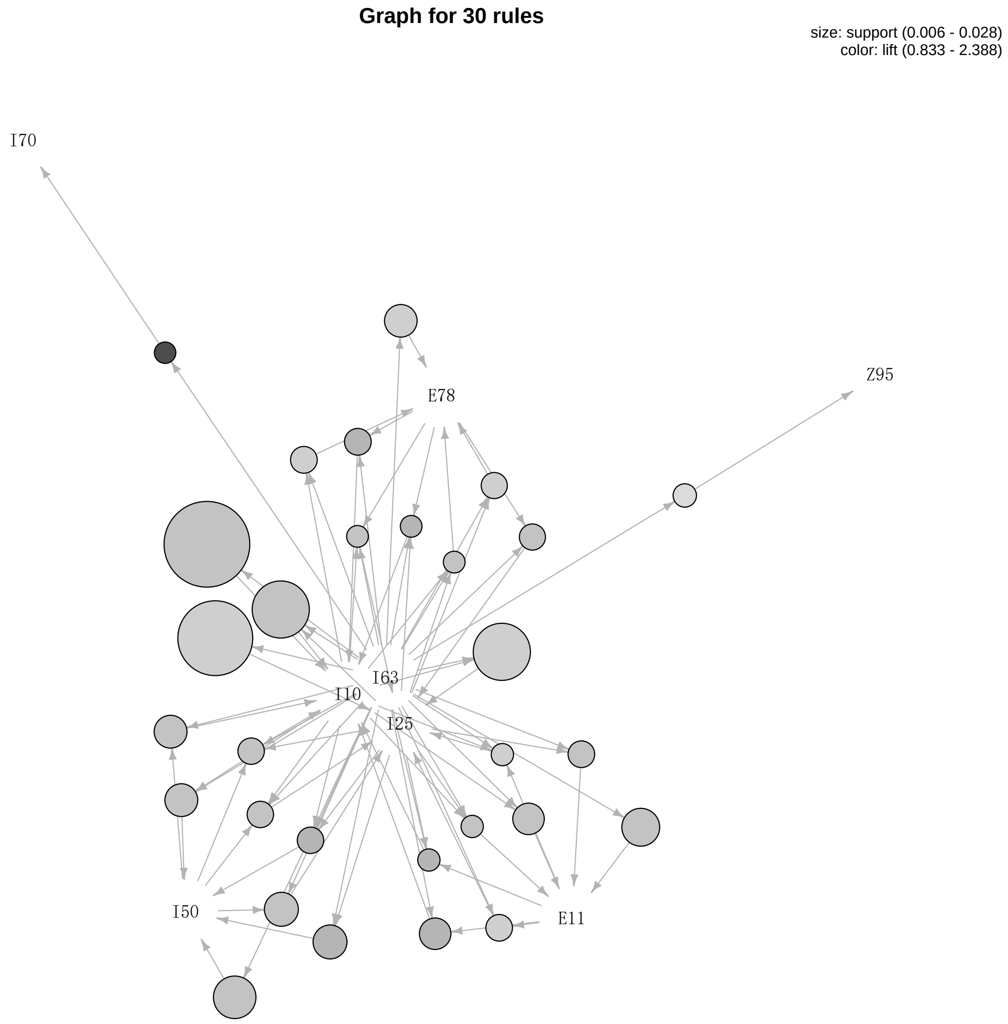

Supplementary figure S2 Identification of comorbid conditions associated with post-procedural ischemic stroke following percutaneous coronary intervention based on association rule mining.

I63: post-procedural ischemic stroke; I10: hypertension; I25: ischemic heart disease; I50: congestive heart failure; E78: hyperlipidemia; E11: diabetes mellitus; I70: Peripheral vascular disease; Z95 Presence of cardiac and vascular implants and grafts.

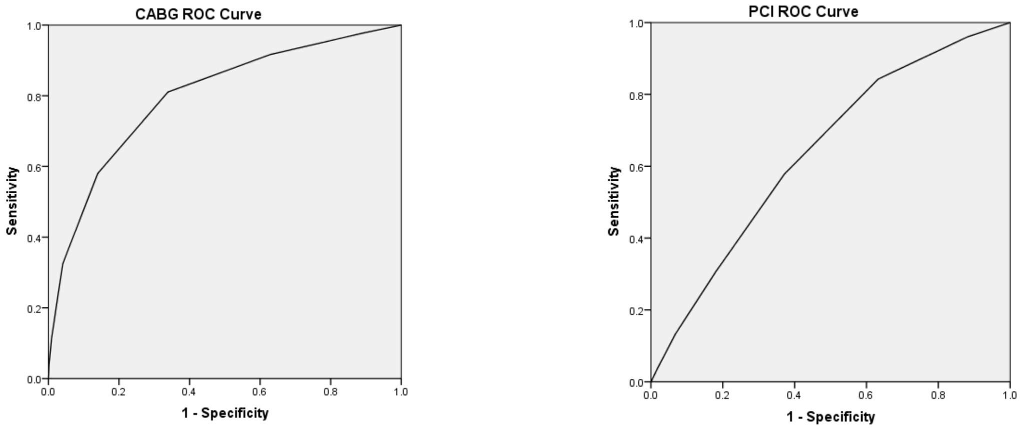

Supplementary figure S3 ROC curves for coronary artery bypass grafting (CABG) and percutaneous coronary intervention (PCI).

Supplementary Table S1 Adjusted odds ratios (ORs) of post-procedural ischemic stroke in patients undergoing coronary artery bypass grafting (CABG) and percutaneous coronary intervention (PCI) stratified by CHA_2DS_2-VASc score.

	Crude OR	95% CI	Adjusted OR[b]	95% CI
CABG				
0	1			
1	1.10	0.59-2.04	1.03	0.56-1.91
≥2	6.96[a]	4.09-11.84	6.28[a]	3.69-10.68
P value for trend	<0.001			
PCI				
0	1			
1	1.42[a]	1.09-1.85	1.42[a]	1.09-1.85
≥2	3.99[a]	3.16-5.05	4.04[a]	3.19-5.11
P value for trend	<0.001			

[a] : $P<0.001$

[b] : Adjusted for a history of hyperlipidemia, chronic obstructive pulmonary disease, ischemic heart disease, and previous cardiac and vascular implants and grafts.
